# Handwritten Digit Recognition with Flood Simulation and Topological Feature Extraction

**DOI:** 10.3390/e27121218

**Published:** 2025-11-29

**Authors:** Rafał Brociek, Mariusz Pleszczyński, Jakub Błaszczyk, Maciej Czaicki, Christian Napoli

**Affiliations:** 1 Department of Artificial Intelligence Modelling, Faculty of Applied Mathematics, Silesian University of Technology, 44-100 Gliwice, Poland; rafal.brociek@polsl.pl; 2 Department of Mathematical Methods in Technology and Computer Science, Faculty of Applied Mathematics, Silesian University of Technology, 44-100 Gliwice, Poland; 3 Faculty of Applied Mathematics, Silesian University of Technology, 44-100 Gliwice, Poland; jb315939@student.polsl.pl (J.B.); mc315940@student.polsl.pl (M.C.); 4 Department of Computer, Control, and Management Engineering, Sapienza University of Rome, Via Ariosto 25, 00185 Roma, Italy; cnapoli@diag.uniroma1.it; 5 Department of Artificial Intelligence, Czestochowa University of Technology, Dabrowskiego 69, 42-201 Czestochowa, Poland

**Keywords:** neural networks, vector database, pattern recognition, classification, water simulation, image recognition, computer vision

## Abstract

This paper introduces a novel approach to handwritten digit recognition based on directional flood simulation and topological feature extraction. While traditional pixel-based methods often struggle with noise, partial occlusion, and limited data, our method leverages the structural integrity of digits by simulating water flow from image boundaries using a modified breadth-first search (BFS) algorithm. The resulting flooded regions capture stroke directionality, spatial segmentation, and closed-area characteristics, forming a compact and interpretable feature vector. Additional parameters such as inner cavities, perimeter estimation, and normalized stroke density enhance classification robustness. For efficient prediction, we employ the Annoy approximate nearest neighbors algorithm using ensemble-based tree partitioning. The proposed method achieves high accuracy on the MNIST (95.9%) and USPS (93.0%) datasets, demonstrating resilience to rotation, noise, and limited training data. This topology-driven strategy enables accurate digit classification with reduced dimensionality and improved generalization.

## 1. Introduction

Handwritten digit recognition remains a foundational problem in computer vision with applications in document analysis, automation, and embedded systems. While deep learning models, particularly convolutional neural networks (CNNs), have achieved near-human accuracy on benchmark datasets like MNIST [1,2,3,4], they often require large training datasets, substantial computational resources, and offer limited interpretability [5,6,7,8,9,10]. These constraints pose challenges in resource constrained or transparency critical settings.

Conventional approaches, including CNNs, focus primarily on pixel-level patterns and local features. However, such methods may overlook global structural cues inherent to digit shapes, such as the loops in ‘8’ or the curved tail of ‘2’, which are intuitive to human perception. Few existing methods are explicitly designed to capture such topological structures.

Over the past two decades, a wide range of methods have been applied to handwritten digit recognition on MNIST, each with different trade-offs in accuracy, interpretability, and computational efficiency. Among classical approaches, *k*-nearest neighbors (*k*-NN) remains a strong baseline due to its simplicity and performance. Applied to raw pixel data, *k*-NN typically achieves 95–97% accuracy [11,12]. With feature engineering such as structural shape descriptors or distance weighted voting, its performance can reach up to 98.5–99% [13] in more complex models. However, *k*-NN’s high memory usage and slow inference on large datasets remain key limitations [14]. Other classical methods have explored structural properties such as concavities to improve discriminative power without increasing model complexity [15].

Support vector machines (SVMs) have shown strong performance with test error rates ranging from 0.6% to 1.4% depending on the kernel and preprocessing techniques used [16,17]. For instance, DeCoste and Schölkopf report 0.56% test error using Gaussian kernels and virtual support vectors [16]. While effective, SVMs are computationally expensive to train on large datasets and often require the manual tuning of hyperparameters [18,19,20,21].

Decision trees offer interpretability and fast training, but often yield subpar results on MNIST due to overfitting and their sensitivity to noise, with accuracies typically below 90% [22]. CNNs dominate the state of the art on MNIST with deep hierarchical feature extraction enabling them to capture spatial and compositional patterns in digit images. LeNet-5 achieved 99.05% accuracy [11,14], and more recent CNNs using regularization methods like DropConnect have reached error rates as low as 0.21% [17,23]. However, CNNs require large training sets, high computational resources, and lack transparency, making them unsuitable for resource-constrained or interpretable machine learning settings.

In this work, we propose a novel feature extraction technique inspired by directional water simulation principles. Treating grayscale images as topographical surfaces, we simulate water propagation from image boundaries using a modified breadth-first traversal. This simulation captures directional and structural characteristics, yielding compact, topology-aware feature vectors that preserve critical shape information while dramatically reducing dimensionality. Unlike black-box neural networks, our approach is interpretable, is efficient, and demonstrates superior performance in low-data scenarios. It enables fast classification using approximate nearest neighbor search with ensemble-based tree partitioning, and it exhibits natural robustness to rotation and noise.

Experiments on the MNIST dataset show that our method achieves accuracy comparable to classical *k*-NN while reducing vector size by over 93% and improving computational efficiency by up to 88%. These results indicate that the proposed method offers a viable alternative for digit recognition in resource-constrained environments or applications requiring transparent, interpretable feature extraction processes. Ensemble-based methods combining neural networks and decision trees have also been explored [24], but they often come with increased complexity and reduced interpretability compared to lightweight, structured feature extraction techniques like ours.

Section 2 describes the considered problem of handwritten digit recognition. Section 3 focuses on a detailed description of the proposed method. The presented Flood-Fill Mechanism algorithm is based on simulating water flooding from four directions of a digit image and employs a modified BFS algorithm. This section provides a step-by-step explanation of the system along with the underlying intuition behind it. Section 4 is dedicated to evaluating the effectiveness of the proposed approach. It presents a comparative analysis of accuracy and computational efficiency in relation to other methods. Additionally, it is demonstrated that the proposed method achieves high accuracy even when trained with a relatively small number of samples. Finally, Section 5 outlines the conclusions and suggests directions for future research. The main goal of this work is to evaluate whether our method (flood-simulation-based feature extraction) can achieve accuracy comparable to classical *k*-NN while significantly reducing feature dimensionality and computational cost.

## 2. Problem Formulation

Handwritten digit recognition remains a fundamental yet challenging task in computer vision [25,26,27,28]. While modern deep learning techniques have achieved impressive accuracy rates, they often struggle in scenarios involving variability in handwriting styles, noise introduced during digitization, and insufficient training data, particularly in domain-specific or low-resource contexts. These factors introduce complexities such as non-uniform strokes, artifacts in input images, and generalization issues.

A notable gap in current approaches lies in their limited exploitation of the structural topology inherent to digit shapes. While CNNs are effective at extracting local features, they do not explicitly model structural aspects such as loops (e.g., in ‘8’) or curved trajectories (e.g., in ‘2’), which are crucial for robust recognition.

To address this, we introduce a novel feature extraction method inspired by water simulation principles implemented using the BFS algorithm. Unlike conventional pixel-based approaches, our method vectorizes digit features to preserve directional and topological continuity. Specifically, digits are treated as binary images, and a Directional Flood-Fill algorithm is employed to extract a sequence of directional vectors representing specific features [29,30,31,32]. These vectors are then used to construct a topology graph that captures both spatial relationships and structural connectivity.

Formally, given a binary digit image $I\in {\left\{0,1\right\}}^{m\times n}$, our algorithm produces a set of vectors

$$V=\{\vec{v_{1}},\vec{v_{2}},\ldots ,\vec{v_{k}}\},$$

where each vector $\vec{v_{i}}$ encodes the direction $\theta_{i}$ and length $l_{i}$ of a feature segment. From this representation, we construct a topology graph G=(V,E), which serves as the basis for digit classification.

This topology-aware representation offers several advantages. It reduces the dimensionality of input data, improves interpretability by making feature relationships explicit, and enhances robustness to partial occlusions and scale variations. Furthermore, the method performs well even with limited training data, making it suitable for applications where large annotated datasets are unavailable.

## 3. Proposed Method—Directional Flood Feature Extraction Algorithm

In this study, we utilize the MNIST (Modified National Institute of Standards and Technology) and USPS (United States Postal Service) datasets, both of which contain grayscale images of handwritten digits. MNIST images are represented as matrices of size 28×28, while USPS images have a resolution of 16×16. For consistency, the pixel intensity values from both datasets were normalized to the range [0,1] and subsequently binarized, where a value of 0 corresponds to a white pixel (background) and 1 denotes a black pixel (foreground). These datasets together provide a diverse benchmark for evaluating digit recognition methods.

To facilitate further processing, a binarization function a(x) is applied to convert grayscale pixels to binary format:

(1) $$a\left(x\right)=\left\{\begin{array}{cc} 0, & \mathrm{if}\;x<n,\\ 1, & \mathrm{if}\;x\geq n, \end{array}\right.$$

where *n* denotes the threshold used for binarization. The resulting binary image $A\in {\left\{0,1\right\}}^{m\times n}$ serves as the input for subsequent analysis.

### 3.1. Directional Flood Simulation for Feature Extraction

#### 3.1.1. Method Overview

**Figure 1:**
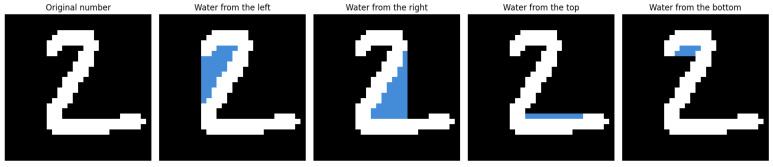
Example of flooding the number ‘2’.

The core idea of the proposed method is to simulate a directional flooding process over the binary image of a digit. Conceptually, this process imitates the behavior of fluid spreading across the image from one of the four boundaries: left, right, top, or bottom. Each flooding direction is considered independently and contributes to the extraction of topological features that are invariant to rotation and resilient to noise. The idea behind the Flood-Fill Mechanism for the digit 2 is illustrated in Figure 1.

#### 3.1.2. Directional Flood Feature Extraction

The simulation employs a modified BFS flood-fill algorithm, where water propagates from selected boundary pixels in a constrained direction (e.g., from the top boundary downward). The algorithm avoids backtracking to prevent redundant visits and enforces directional consistency.

For each flooding direction, the algorithm records geometric and structural characteristics of the flooded area, such as coverage ratio and boundary complexity. Aggregating the outcomes from all four directions yields a compact, rotation-invariant descriptor of the digit’s topology.

#### 3.1.3. Advantages of the Proposed Approach

- The flooding process mimics natural fluid behavior, effectively capturing topological structures such as loops (as in the digit ‘8’) and open gaps (as in ‘9’).
- Multi-directional flooding reduces sensitivity to rotation and minor distortions.
- The BFS-based implementation is computationally efficient with time complexity linear in the number of image pixels.

### 3.2. Directional Flood Feature Extraction Algorithm

Let $A\in {\left\{0,1\right\}}^{m\times n}$ be the binary input image, where 0 represents a pixel to be filled (background), and 1 represents a blocking pixel (foreground). For a given flooding direction s∈{left,right,top,bottom}, the algorithm generates a filled matrix *F* as follows:

1. Initialization
  - F←A                        // copy of the original matrix;
  - Q← queue of initial seed pixels;
  - V← visitation matrix (initialized to false).
2. Seed Selection
  (2) $$Q\leftarrow \left\{\begin{array}{cc} \{\left(i,1\right)|A_{i,1}=0\}, & \mathrm{if}\;s=\mathrm{left},\\ \{\left(i,n\right)|A_{i,n}=0\}, & \mathrm{if}\;s=\mathrm{right},\\ \{\left(1,j\right)|A_{1,j}=0\}, & \mathrm{if}\;s=\mathrm{top},\\ \{\left(m,j\right)|A_{m,j}=0\}, & \mathrm{if}\;s=\mathrm{bottom}. \end{array}\right.$$
  All selected seed pixels are marked as visited in *V*.
3. Flood Propagation
  (a) While Q≠∅:
    i.   Dequeue (i,j)←Q,
    ii.  Set $F_{i,j}\leftarrow 1,$                          // fill pixel
    iii. Determine neighboring positions $\left(i^{\prime },j^{\prime }\right)$ based on the allowed flood direction:
      (3) $$N\leftarrow \left\{\begin{array}{cc} \{(i\pm 1,j),(i,j\pm 1)\}, & \mathrm{if}\;\mathrm{backtracking}\;\mathrm{is}\;\mathrm{allowed},\\ \{(i\pm 1,j),(i,j+d)\}, & \mathrm{otherwise}, \end{array}\right.$$
      where d=1 (right) for left-side flooding, d=−1 (left) for right-side flooding, etc.
    iv. For each neighbor $(i^{\prime },j^{\prime })\in N$, if the following apply:
      - $\left(i^{\prime },j^{\prime }\right)$ is within bounds,
      - $V_{i^{\prime },j^{\prime }}=\mathrm{false}$,
      - $F_{i^{\prime },j^{\prime }}=0$,
      - and (if no backtracking) the move is away from the origin side,
      then:
      - Enqueue $(i^{\prime },j^{\prime })\to Q$,
      - Mark $V_{i^{\prime },j^{\prime }}\leftarrow \mathrm{true}$.
4. **Output**: Return the filled matrix *F*.

**Figure 2:**
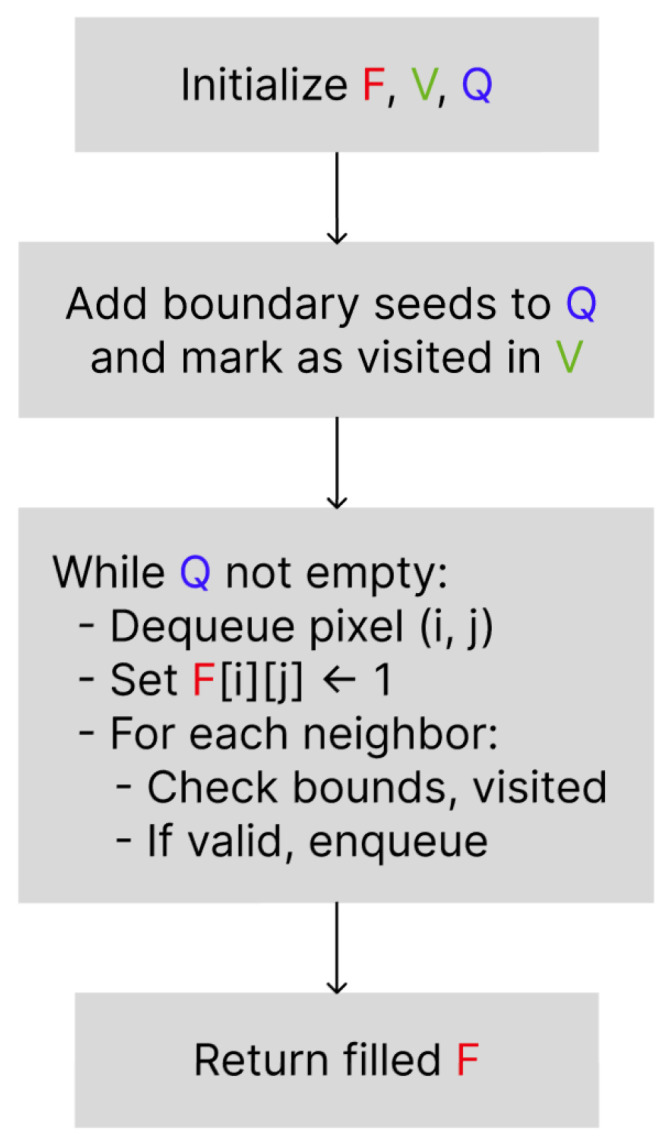
High-level flowchart of the Directional Flood-Fill algorithm starting from the image boundary.

Figure 2 presents the block diagram of the proposed Directional Flood Feature Extraction algorithm.

### 3.3. Segmentation

**Figure 3:**
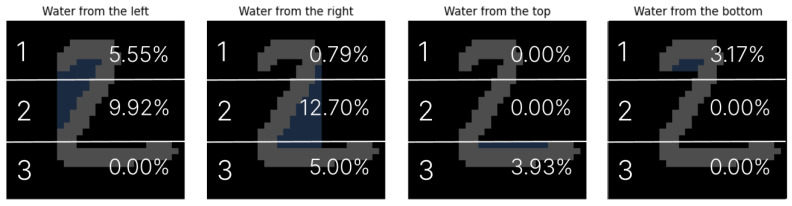
Illustrative segmentation for N=3; numerical values may not reflect actual measurements.

Each digit image is vertically partitioned into *N* equal segments, and the proportion of the flooded area is computed for each segment individually. The resulting values are aggregated into a feature vector that characterizes the directional water distribution across the digit. The concept of this mechanism is illustrated in Figure 3.

For example, for the digit ‘2’ and a division into N=3 segments, the feature vector takes the form (for illustrative purposes, the values are rounded to two decimal places):

$$\vec{V_{0}}=\left[0.06,0.1,0.0,0.01,0.13,0.05,0.0,0.0,0.04,0.03,0.0,0.0\right].$$

### 3.4. Extension of the Method for Digits with Enclosed Regions

**Figure 4:**
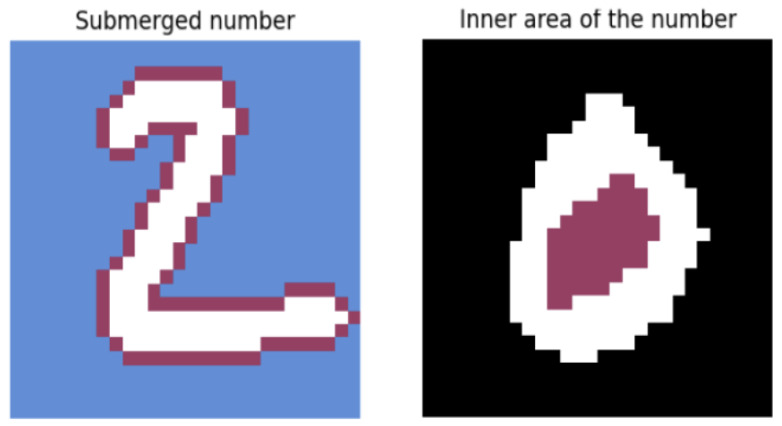
Illustration of the water-fill extension for enclosed regions in digits.

Certain digits, such as ‘0’, ‘6’, and ‘8’, contain enclosed regions that cannot be reached by water in the standard flood-fill simulation. To account for this limitation and enhance the descriptive power of the feature set, three additional parameters were introduced:

1. Inner Area Simulation: To detect the internal regions of the digit, an additional iteration of the BFS algorithm was performed with backward propagation enabled. This simulates water being poured inside the enclosed components of the digit. A graphical representation of this concept is provided in Figure 4. The resulting flooded region is divided into *N* horizontal segments, and the percentage of filled pixels is calculated for each segment.
2. Digit Perimeter Estimation: The second parameter quantifies the degree to which the digit is surrounded by water. This is achieved by simulating the digit’s immersion in a water basin and computing its perimeter. The process is formalized as follows:
  Let $A\in {\left\{0,1\right\}}^{m\times n}$ be the binary matrix representing the digit, and let $B\in {\left\{0,1\right\}}^{m\times n}$ represent the enclosed regions identified in the previous step. The combined matrix *C* is defined as
  $$C_{i,j}=A_{i,j}\cup B_{i,j},\;\mathrm{for}\;1\leq i\leq m,\;1\leq j\leq n.$$
  The normalized perimeter *P* is computed as
  (4) $$P=\frac{1}{m\cdot n}\sum_{i=1}^{m}\sum_{j=1}^{n}1_{C_{i,j}=1}\cdot \left(\sum_{\left(k,l)\in N(i,j\right)}1_{C_{k,l}=0}\right),$$
  where the following apply:
  - N(i,j)={(i−1,j),(i+1,j),(i,j−1),(i,j+1)} denotes the set of 4-connected neighbors (excluding out-of-bounds positions),
  - $1_{\mathrm{condition}}$ is the indicator function, which is equal to 1 if the condition is satisfied and 0 otherwise,
  - the result is normalized by the total number of pixels in the matrix, m·n.
  A graphical representation of this concept is provided in Figure 4.
3. Segmented Pixel Density: As the third feature, the original binary digit matrix is divided into *N* horizontal segments. In each segment, the number of digit pixels is counted and normalized by the total number of pixels in that segment, yielding a measure of pixel density.

The features derived from the three steps above are concatenated to form an extended feature vector. For example, for digit ‘2’ and division into N=3 segments, the resulting feature vector is shown below:

$$\vec{V_{1}}=\left[2.0,0.06,0.1,0.0,0.01,0.13,0.05,0.0,0.0,0.04,0.03,0.0,0.0,0.0,0.0,0.0,0.1,0.13,0.22,0.1\right].$$

Note that the first element corresponds to the digit label and is used during the classification phase.

**Remark 1.** 
*In the final implementation, values were computed for segmentations into N=5 and N=7, and feature values were rounded to 17 decimal places. The above example with N=3 and rounding to two decimal places is presented solely for illustrative purposes.*

### 3.5. Classification Phase

The classification procedure is based on comparing an input feature vector with a set of reference vectors generated during the training phase. Each component of the feature vector defines a dimension in a high-dimensional feature space. In this context, the proximity of vectors reflects similarity in the corresponding characteristics. For example, the Inner Area metric exhibits reduced discriminative power between certain digit pairs, such as 0 and 6.

To enhance computational efficiency, the classical *k*-NN algorithm was replaced with the Approximate Nearest Neighbors Oh Yeah (Annoy) method, which provides faster querying in high-dimensional spaces while preserving acceptable accuracy.

#### 3.5.1. Hierarchical Partitioning via Binary Trees

The method employs recursive binary space partitioning using randomly constructed trees. This method organizes the high-dimensional vector space into a hierarchical structure, enabling efficient nearest-neighbor retrieval via distance-based tree traversal. The algorithm integrates randomized node selection with ensemble aggregation to balance classification accuracy and computational efficiency.

At each internal node, two reference vectors, $r_{\mathrm{left}}$ and $r_{\mathrm{right}}$, are selected at random. The remaining vectors are partitioned into two disjoint subsets based on their Euclidean distance to these reference vectors:

(5) $$v\in \left\{\begin{array}{cc} S_{\mathrm{left}}, & \mathrm{if}\;{∥v-r_{\mathrm{left}}∥}_{2}<{∥v-r_{\mathrm{right}}∥}_{2},\\ S_{\mathrm{right}}, & \mathrm{otherwise}, \end{array}\right.$$

where ${∥\cdot ∥}_{2}$ denotes the Euclidean norm. To avoid highly imbalanced splits that could lead to degenerate tree structures, a balance condition is enforced. A split is retained only if the resulting partition satisfies the following:

(6) $$\frac{|S_{\mathrm{left}}|}{|S_{\mathrm{left}}|+|S_{\mathrm{right}}|}\in \left(1-\eta ,\eta \right),$$

where η∈(0.5,1) controls the acceptable level of imbalance. In this study, a value of η=0.95 was empirically selected, allowing for relatively unbalanced partitions while maintaining logarithmic tree depth. Specifically, the worst-case depth is bounded by $𝒪(\log_{1/\varepsilon }N)$, where ε=1−η (e.g., $\log_{20}N$ for η=0.95).

#### 3.5.2. Ensemble Forest Construction

To mitigate variance introduced by the stochastic nature of tree construction, an ensemble of *N* independent binary trees is built. Each tree is initialized using a distinct random seed, resulting in a diverse set of hierarchical partitions. This ensemble strategy increases the robustness of the classifier and reduces the risk of overfitting associated with individual trees. Final predictions are aggregated across all trees using a consensus-based mechanism.

#### 3.5.3. Querying Process

During inference, a query vector *q* is independently traversed through each tree in the ensemble. At each internal node *N*, the next node is selected according to the following decision rule:

(7) $$N_{\mathrm{next}}=\left\{\begin{array}{cc} N_{\mathrm{left}}, & \mathrm{if}\;∥q-r_{\mathrm{left}}{∥}_{2}<{∥q-r_{\mathrm{right}}∥}_{2}-\tau ,\\ N_{\mathrm{right}}, & \mathrm{if}\;∥q-r_{\mathrm{left}}{∥}_{2}>{∥q-r_{\mathrm{right}}∥}_{2}+\tau ,\\ N_{\mathrm{left}}\;\mathrm{and}\;N_{\mathrm{right}}, & \mathrm{otherwise}, \end{array}\right.$$

where τ is a predefined tolerance threshold used to handle ambiguous decisions near the midpoint between reference vectors.

Upon reaching a leaf node, the standard *k*-NN algorithm is applied (see Section 4.3), which is restricted to the vectors contained within that node. This localized comparison ensures fast, approximate nearest-neighbor retrieval with controllable precision.

#### 3.5.4. Label Assignment via Majority Voting

The predicted class label $\hat{l}$ is determined via majority voting among the labels retrieved via *k*-NN across across all trees. Let $L=\{l_{1},l_{2},\ldots ,l_{k}\}$ denote the set of candidate labels. The final classification is given by the following:

(8) $$\hat{l}=\underset{l\in L}{\arg\;\max}\sum_{i=1}^{k}I\left(l_{i}=l\right),$$

where I(·) denotes the indicator function. This ensemble-based voting mechanism enhances generalization performance and reduces sensitivity to outliers or individual misclassifications.

**Figure 5:**
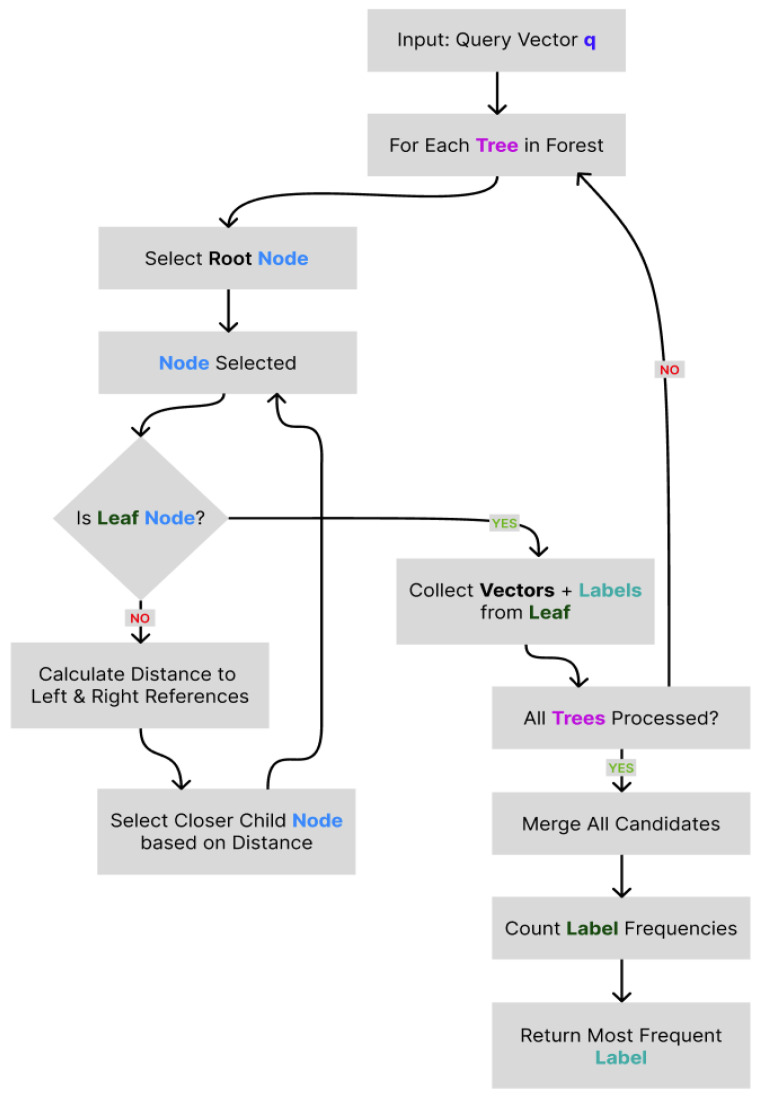
Flowchart of Annoy querying process.

Figure 5 shows the block diagram of the classification procedure. The diagram outlines the sequential steps involved in traversing the binary tree ensemble, collecting candidate vectors, and aggregating results. It provides a schematic overview of the decision process leading to the final label assignment.

## 4. Results

This section presents the results obtained using the proposed handwriting recognition algorithm. A series of tests was conducted to demonstrate the effectiveness of the method.

### 4.1. Methodology

For the experiments, we used two benchmark datasets: MNIST and USPS. The MNIST dataset consists of 60,000 training images and 10,000 testing images, while the USPS dataset contains 7291 training images and 2007 testing images. In both cases, the testing set is distinct from the training set, ensuring that the algorithm is not overfitted to the training data. For each test sample, the prediction algorithm was executed, and the result was compared with the actual label.

Accuracy was calculated using the formula

$$\mathrm{Accuracy}=\left(\frac{\mathrm{correct}\;\mathrm{predictions}}{\mathrm{total}\;\mathrm{test}\;\mathrm{samples}}\right)\times 100\%,$$

where the total number of test samples was 10,000 for MNIST and 2007 for USPS.

All tests were conducted on a computer with the following specifications: CPU: i5-12540H, RAM: 16 GB 3200 MHz, GPU: Nividia RTX 3050 Ti mobile.

### 4.2. Performance Comparison Across Initial Parameters

The study presents several initial parameters, including the binarization threshold value, matrix segmentation number, and the number of trees used in classification. Adjusting these parameters yields differing final results. During the evaluation of the proposed method, optimal values were identified to balance satisfactory accuracy, vector dimensionality, and prediction runtime.

For the matrix segmentation parameter, it was observed that odd-numbered partition sizes consistently achieved higher accuracy compared to even-numbered values. To maintain a compact vector length, segmentation sizes of 5 and 7 were selected. For odd-sized segments, the algorithm tends to perform better because the center aligns naturally with a single pixel, avoiding ambiguity in path splitting or merging during propagation. In contrast, even-sized segments introduce a symmetrical boundary that passes between pixels, which can lead to additional branching or uncertainty in how the flood-fill expands, potentially causing inaccuracies or artifacts at segment borders.

**Figure 6:**
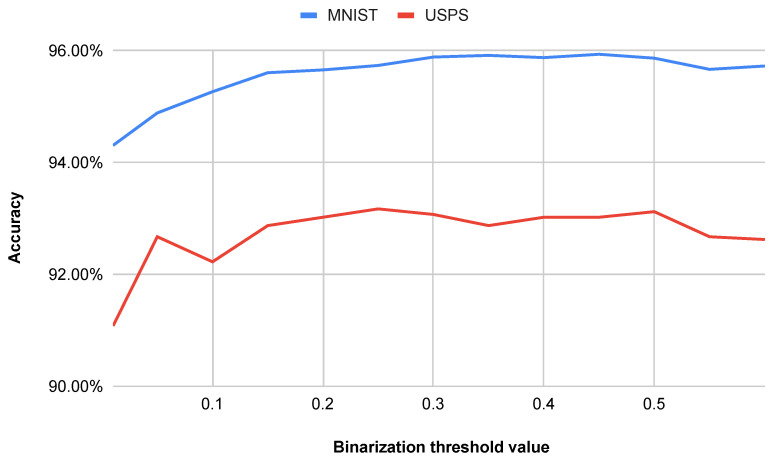
Impact of the binarization threshold value on accuracy.

The binarization threshold was observed to have a minor effect on the classification accuracy, with variations of approximately 1% for the MNIST dataset and around 3% for the USPS dataset, the latter being slightly higher due to its increased noise. Empirically, the accuracy reached a maximum at a threshold value of around 0.30 for both datasets. Consequently, this value was selected for subsequent experiments. Pixels with intensity values exceeding 30% of the maximum possible intensity were assigned a value of 1, while all other pixels were assigned a value of 0. The relationship between the binarization threshold and the classification accuracy is illustrated in Figure 6, where it can be seen that deviations from the optimal threshold lead to a gradual decrease in accuracy.

**Figure 7:**
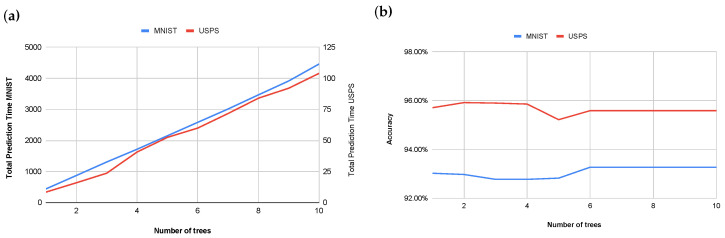
Impact of tree count on computational time (**a**) and impact of tree count on model accuracy (**b**).

Figure 7a,b illustrate the relationship between the number of trees used in the ensemble model and both computational cost and classification accuracy. An examination of these results reveals that increasing the number of trees beyond two yields negligible improvements in model accuracy while concurrently leading to a consistent increase in computational cost. An initial increase from one to two trees demonstrated a marginal accuracy gain of 0.21 percentage points on MNIST (from 95.71% to 95.92%) while no improvement was observed on the USPS dataset. Subsequent increases in the number of trees did not produce statistically significant or consistent improvements in accuracy. Indeed, certain configurations exhibited a slight reduction in accuracy, for example, on MNIST a decrease from 95.22% at two trees to 95.22% at five trees, and on USPS a decrease from 92.97% at two trees to 92.83% at five trees. From six to ten trees, the model accuracy plateaued at 95.62% on MNIST and 94.92% on USPS. This observed plateau in accuracy, despite increasing model complexity, suggests the potential for overfitting. Therefore, a value of two trees was selected for the ensemble model. This decision was based on the empirical observation that higher tree counts did not yield a perceptible improvement in prediction accuracy while concurrently resulting in a linear increase in computational prediction time. This approach thus optimizes for computational efficiency without a noticeable compromise in overall model performance.

### 4.3. Benchmarking Against *k*-NN

Two best-performing configurations of our method were compared to a baseline *k*-NN classifier. The *k*-NN algorithm used for comparison was implemented according to the description in [33]. Table 1 presents a comparison of the *k*-NN method with the approach proposed in this paper. The results of the tests on the USPS dataset are presented in Table 2.

On the full MNIST and USPS datasets, the proposed method demonstrates two significant advantages over *k*-NN:

1. Dimensionality reduction with minimal accuracy loss (best *k*-NN accuracy was taken):
  - For N = 5, our method reduces the feature vector length from 784 (*k*-NN) to 31 (96% reduction), while the accuracy decreases by only 1.73%.
  - For N = 7, the vector length is reduced to 43 (94.5% reduction) with an accuracy drop of 1.43%.
2. Substantial computational efficiency:
  - Compared to *k*-NN (Euclidean), our approach is 74.0% faster (888.53 s vs. 3422.08 s) on the MNIST dataset and 77.3% faster (15.99 s vs. 70.34 s) on the USPS dataset.
  - Compared to *k*-NN (Cosine), our approach is 86.2% faster (888.53 s vs. 6448.41 s) on the MNIST dataset and 87.9% faster (15.99 s vs. 132.74 s) on the USPS dataset.

**Figure 8:**
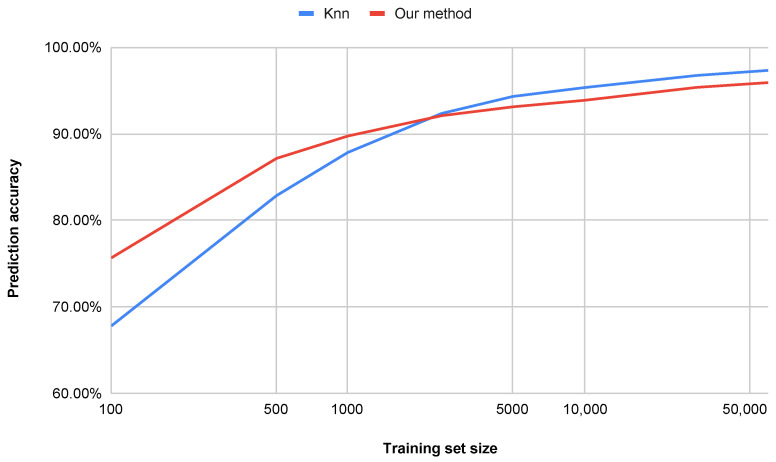
Comparison of achieved accuracy with respect to the number of training set samples.

**Figure 9:**
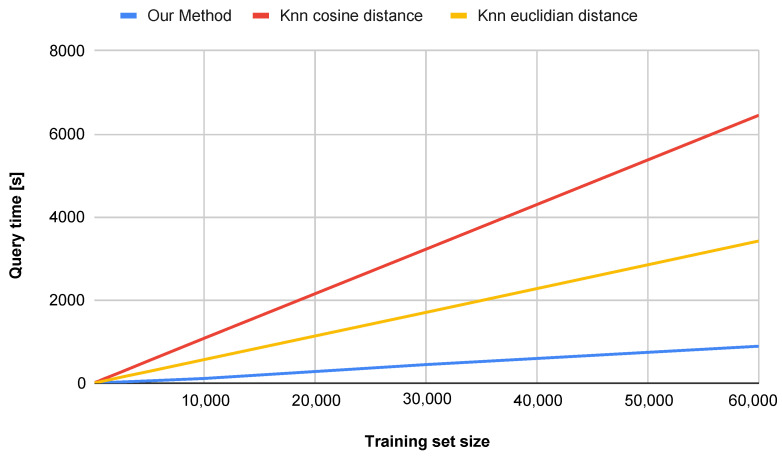
Comparison of query time with respect to the number of training set samples.

An additional advantage of the proposed method is its superior performance in terms of computational efficiency, dimensionality reduction, and accuracy on datasets smaller than 60,000 samples. Other approaches that achieve similar accuracy are often far more complex and less interpretable [34,35], while our approach remains efficient and transparent. Figure 8 illustrates the impact of the number of training set samples on the classification accuracy for both the *k*-NN method and our proposed approach (water simulation). For smaller datasets (below approximately 20,000 samples), the water simulation method achieves higher accuracy. For datasets larger than 20,000 samples, our method yields slightly lower, yet still comparable, accuracy. It is worth noting, however, that the computation time is significantly shorter, as shown in Figure 9.

**Figure 10:**
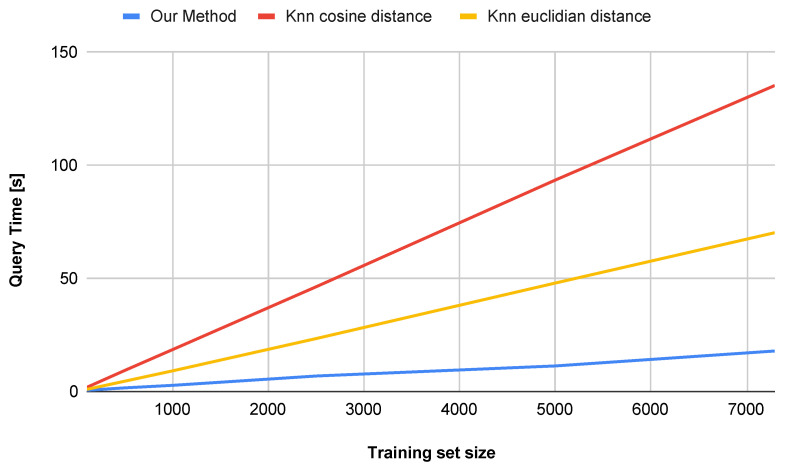
Comparison of query time with respect to the number of training set samples on the USPS dataset.

Key observations are summarized below:

1. Accuracy vs. Dataset Size (Figure 8):
  - The proposed method (water simulation) maintains consistently high accuracy, even with small training sets (e.g., achieving strong performance with only 500 samples), whereas the *k*-NN method requires more than 1100 samples to reach comparable accuracy.
  - With a training set of 100 samples, our approach outperforms *k*-NN by 10.8 percentage points, indicating superior generalization capability in low-data regimes.
2. Query Time vs. Dataset Size (Figure 9 and Figure 10):
  - Our method demonstrates sub-linear runtime scaling as the dataset size increases, in contrast to the *k*-NN method (both Euclidean and Cosine similarity variants), which exhibits near-quadratic growth.
  - At 10,000 samples, our method achieves a runtime reduction of 86.2% compared to *k*-NN with Cosine similarity and 74.0% compared to *k*-NN with Euclidean distance.

Analysis of the confusion matrices generated for the two datasets provides critical insight into the classification behavior of the model. The confusion matrix for the MNIST test set (see Table 3) reveals that the most prominent misclassifications occur between the following three digit pairs: 3 and 5; 9 and 4; and 7 and 2. A similar trend is observed in the confusion matrix derived from the USPS dataset (see Table 4). Although the specific error counts differ due to dataset variability, the model again exhibits confusion between visually similar classes, indicating that the misclassification patterns are consistent across both datasets. This consistency suggests that the model’s limitations stem not only from dataset-specific properties but also from inherent ambiguities in distinguishing certain handwritten digits.

The common misclassification between digit 3 and digit 5 (and vice versa) can be attributed to the similarity in their extracted features, specifically patterns related to water accumulation in horizontal segments corresponding to digit indents. Although water collects on the left side for digit 3 and on the right for digit 5, the resulting feature vectors are sufficiently similar to induce classification errors. Misclassifications between digit 9 and digit 4 frequently arise when the rendered or input digit 9 lacks a clearly defined rounded bottom. In such instances, the water distribution across the segments closely resembles that of the digit 4. Comparing digits 7 and 2, it is observed that they retain similar amounts of water when analyzed from the right, left, and top perspectives. When the bottom part of digit 2 is poorly defined, misclassification errors in categorization can arise.

## 5. Conclusions

In this paper, we proposed a novel water simulation-based approach to handwritten digit recognition, leveraging a directional flood-fill algorithm to extract meaningful structural and topological features. We compared our method with traditional *k*-NN using both Euclidean and cosine distances, observing comparable accuracy while reducing feature dimensionality by over 93% and improving computational efficiency by up to 88%. The algorithm is intuitive, interpretable, and particularly effective in low-data scenarios. Key strengths include robustness to noise and rapid computation.

While the current results are promising, several directions for future enhancement remain. These include incorporating 2D (grid-like) segmentation, applying differential weighting to feature components, aggregating outputs from multiple runs to increase robustness and the implementation of multithreading to improve computational efficiency. Importantly, since the algorithm can be trained on arbitrary shapes, its use is not limited to numbers and letters but rather may be extended to a broad range of monochrome images, underscoring its versatility in general shape-based recognition tasks. Overall, this approach offers a versatile and efficient framework for pattern recognition that may extend far beyond digit classification.

## Figures and Tables

**Table 1:** Comparison of classification accuracy between our method and the *k*-NN method on the MNIST datset.

Our Method and *k*-NN Comparison on MNIST Dataset				
Method	Vec. Length	Train (s)	Query (s)	Acc. (%)
Our Method (N = 5)	31	6.32	800.26	94.9
Our Method (N = 7)	43	6.69	888.53	95.9
Our Method (N = 9)	55	7.28	989.17	96.4
*k*-NN Euclidean (K = 3)	784	n/a	3422.08	97.1
*k*-NN Euclidean (K = 5)	784	n/a	3442.98	96.9
*k*-NN Euclidean (K = 7)	784	n/a	3447.05	96.9
*k*-NN Cosine (K = 3)	784	n/a	6448.41	97.3
*k*-NN Cosine (K = 5)	784	n/a	6314.58	97.3
*k*-NN Cosine (K = 7)	784	n/a	6284.65	97.3

**Table 2:** Comparison of classification accuracy between our method and the *k*-NN method on the USPS datset.

Our Method and *k*-NN Comparison on USPS Datset				
Method	Vec. Length	Train (s)	Query (s)	Acc. (%)
Our Method (N = 5)	31	0.72	14.92	91.9
Our Method (N = 7)	43	0.73	15.99	93.0
Our Method (N = 9)	55	0.76	18.09	93.3
*k*-NN Euclidean (K = 3)	784	n/a	70.22	94.5
*k*-NN Euclidean (K = 5)	784	n/a	70.34	94.5
*k*-NN Euclidean (K = 7)	784	n/a	69.64	94.2
*k*-NN Cosine (K = 3)	784	n/a	135.32	94.2
*k*-NN Cosine (K = 5)	784	n/a	132.74	94.2
*k*-NN Cosine (K = 7)	784	n/a	135.19	93.8

**Table 3:** Confusion matrix for MNIST.

	Predicted	0	1	2	3	4	5	6	7	8	9
Actual											
0		971	0	1	0	2	1	2	3	0	0
1		0	1130	2	1	0	0	2	0	0	0
2		4	1	996	2	7	2	10	9	1	0
3		0	2	7	958	0	26	0	15	2	0
4		1	2	0	0	948	0	7	0	0	24
5		3	8	2	20	0	837	8	5	3	6
6		5	8	1	0	7	1	934	0	1	1
7		0	9	14	4	2	2	0	995	0	2
8		14	4	4	4	9	2	10	3	916	8
9		6	9	0	4	19	2	0	13	10	946

**Table 4:** Confusion Matrix for USPS.

	Predicted	0	1	2	3	4	5	6	7	8	9
Actual											
0		352	0	3	1	1	0	1	1	0	0
1		0	258	0	0	2	0	3	0	0	1
2		5	1	183	2	5	0	1	1	0	0
3		2	0	5	148	0	10	0	0	1	0
4		3	6	2	0	180	0	1	0	0	8
5		5	0	2	5	0	140	1	0	4	3
6		2	0	1	0	3	0	164	0	0	0
7		0	1	2	0	5	0	0	136	0	3
8		7	3	0	0	1	1	3	1	143	7
9		1	0	1	0	3	0	0	1	2	169

## Data Availability

Data is contained within the article.
